# Tuning Optical and Electrical Properties of Vanadium Oxide with Topochemical Reduction and Substitutional Tin

**DOI:** 10.1021/acs.chemmater.4c01557

**Published:** 2024-10-17

**Authors:** Lance M. Wheeler, Thanh Luan Phan, Michelle A. Smeaton, Swagata Acharya, Shruti Hariyani, Marlena E. Alexander, Miranda I. Gonzalez, Elisa M. Miller, David W. Mulder, Sarbajit Banerjee, Katherine L. Jungjohann, Andrew J. Ferguson, Jeffrey L. Blackburn

**Affiliations:** † National Renewable Energy Laboratory, Golden, Colorado 80401, United States; ‡ Department of Chemistry, Texas A&M University, College Station, Texas 77843, United States

## Abstract

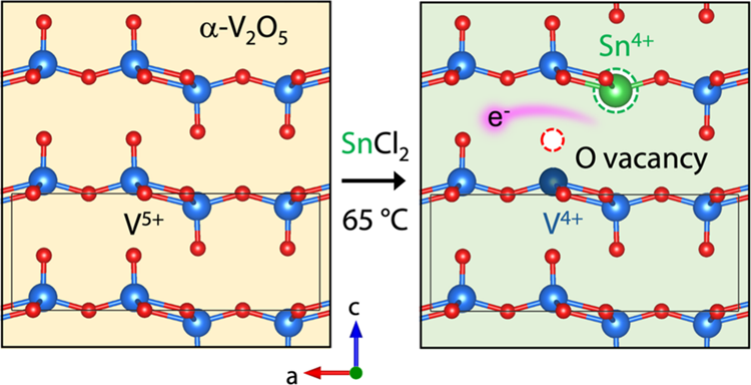

Vanadium oxides are widely tunable materials, with many thermodynamically stable phases suitable for applications spanning catalysis to neuromorphic computing. The stability of vanadium in a range of oxidation states enables mixed-valence polymorphs of kinetically accessible metastable materials. Low-temperature synthetic routes to, and the properties of, these metastable materials are poorly understood and may unlock new optoelectronic and magnetic functionalities for expanded applications. In this work, we demonstrate topochemical reduction of α-V_2_O_5_ to produce metastable vanadium oxide phases with tunable oxygen vacancies (>6%) and simultaneous substitutional tin incorporation (>3.5%). The chemistry is carried out at low temperature (65 °C) with solution-phase SnCl_2_, where Sn^2+^ is oxidized to Sn^4+^ as V^5+^ sites are reduced to V^4+^ during oxygen vacancy formation. Despite high oxygen vacancy and tin concentrations, the transformations are topochemical in that the symmetry of the parent crystal remains intact, although the unit cell expands. Band structure calculations show that these vacancies contribute electrons to the lattice, whereas substitutional tin contributes holes, yielding a compensation doping effect and control over the electronic properties. The SnCl_2_ redox chemistry is effective on both solution-processed V_2_O_5_ nanoparticle inks and mesoporous films cast from untreated inks, enabling versatile routes toward functional films with tunable optical and electronic properties. The electrical conductance rises concomitantly with the SnCl_2_ concentration and treatment time, indicating a net increase in density of free electrons in the host lattice. This work provides a valuable demonstration of kinetic tailoring of electronic properties of vanadium–oxygen systems through top-down chemical manipulation from known thermodynamic phases.

## Introduction

The family of vanadium oxides receives immense interest due to its exceptional tunability of properties using stoichiometry,^1^ crystal structure,^2^ and doping.^3^ Despite the rich history, the community continues to develop new insights into these materials, and they are considered a prototypical platform for applications related to the control and dynamic modulation of their optical and electronic properties. Various vanadium–oxygen stoichiometries have been explored for technologies ranging from optical modulators and electrochromic devices (i.e., dynamic windows) to nonlinear conductance switching (i.e., for memory, neuromorphic computing, or sensing).^4^ Electrochemical energy storage is a particularly promising area, with a range of ion intercalation chemistries demonstrated.^5^ Attractive features of vanadium oxides include the rich compositional space (phase diagram), associated variations of the crystal structure, a broad range of vanadium redox and spin states, and a correspondingly rich swath of electronic properties (spanning the range from insulator to semiconductor to metallic).^2,6^

The stability of many different oxidation states in vanadium oxides allows for many different polymorphs. Wadsley phases are a family of vanadium oxides, known to include V_2_O_5_, V_3_O_7_, V_4_O_9_, and V_6_O_13_, which correspond to a general formula: V_*n*_O_2*n*+1_, where *n* = 2–6.^4^ The most oxidized compound V_2_O_5_ contains vanadium solely in the V^5+^ state. α-V_2_O_5_ is layered and is known to accommodate heterovalent atoms, either interstitially within the quasi-van der Waals gap or substitutionally at metal sites within the lattice. Interstitial cations from group 1 alkali metal (e.g., Li^+^, Na^+^, K^+^) or group 2 alkaline earth metals (e.g., Mg^2+^, Ca^2+^) are routinely incorporated after synthesis using chemical or electrochemical methods.^4^ Conversely, several transition metal (e.g., Ti^4+^, Mn^4+^, Mo^6+^) and post-transition metal (e.g., Sn^4+^, Al^3+^, Bi^3+^) ions have been incorporated as substitutional dopants into the α-V_2_O_5_ lattice.^7−16^ Though examples of substitutional doping are prevalent, there are only a few reports of single-crystal or high-resolution structural data.^14,16^ Thermodynamically stable phases of vanadium oxide are typically synthesized at high temperatures (>400 °C), and substitutional incorporation of heterovalent atoms is typically achieved during synthesis via sol–gel, hydrothermal/solvothermal, or solid-state synthetic routes at high temperature and/or pressures.^8,11−16^ Examples of low-temperature post-synthesis methods for substitutional doping of the α-V_2_O_5_ structure have not been reported.

Vanadium can be reduced to form metastable oxygen vacancies within the vanadium oxide lattice. Vacancies are typically introduced using harsh chemical conditions like annealing at high temperatures in reducing (H_2_) or inert environments.^17^ There has been recent attention on topotactic (topochemical) transitions of vanadium oxide bronzes to achieve new polymorphs or altered ordering of interstitial sites in the binary vanadium oxide system.^2,18,19^ Topotactic transitions are kinetically controlled structural phase changes accomplished by the ordered loss, gain, or rearrangement of atoms while maintaining the same crystallographic frameworks as the parent phase.^20^ In addition to new binary phases of vanadium oxide, topochemical reduction reactions can also be leveraged to engender site-selective modification, modulate spin- and charge-ordering patterns, continuously expand the crystal lattice, avoid compensation reactions, and create nonequilibrium quantities of oxygen vacancies.^18,21,22^ Topochemical reduction has been demonstrated in many transition metal oxide perovskite systems such as nickelates,^23−25^ manganites,^26,27^ ferrites,^28,29^ and titanates^30,31^ using a solid-state reaction with common reducing agents like CaH_2_, LiH, and NaH, which yields oxygen vacancies in the host oxide lattice that are charge-balanced by protons.

In this work, we introduce the topochemical reduction and substitutional tin incorporation of α-V_2_O_5_ with solution-phase SnCl_2_ at low temperatures (<65 °C). The low-temperature approach yields a metastable vanadium oxide material with oxygen vacancies and substitutional tin while maintaining the same crystal symmetry and average structure as those of the starting α-V_2_O_5_ material. The extents of vanadium reduction and tin doping are tunable using SnCl_2_ concentration and reaction time, which yields control over the optical and electrical properties of vanadium oxide nanoparticles, both in solution and in mesoporous V_2_O_5_ nanoparticle films. As we increase the concentration of SnCl_2_ (0–0.10 M) or extend the treatment duration (0 to 90 min), we observe a corresponding rise in the concentration of oxygen vacancies and concomitant V^4+^ and Sn^4+^ in the lattice. All-electron quasiparticle self-consistent (QS) GW method calculations confirm that vacancies contribute free electrons to the lattice, whereas Sn^4+^ contributes holes. The tunable chemistry maps to a correspondingly tunable density of free electrons and electrical conductance in mesoporous α-V_2_O_5_ thin films, which we track with both contactless (9 GHz) and two-terminal device conductance measurements. This study presents a significant demonstration of tailoring the vanadium–oxygen system through top-down chemical manipulation. Such advancements hold promise for next-generation optoelectronic applications.

## Results and Discussion

### Low-Temperature SnCl_2_ Chemistry

**Figure 1:**
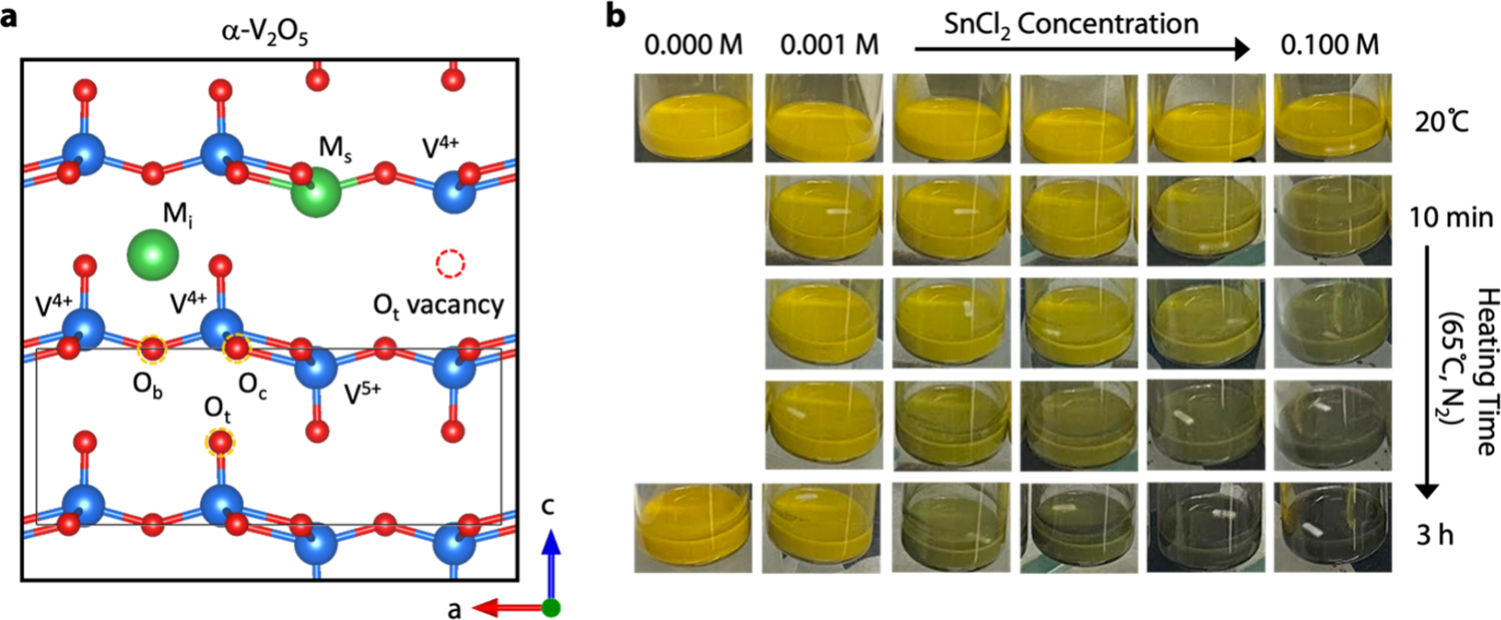
Chemical evolution of V_2_O_5_ from SnCl_2_ redox chemistry. (a) Visualization of the lattice structure of α-V_2_O_5_ based on ICSD 60767. Thin black box highlights the α-V_2_O_5_ unit cell. O_t_ = terminal oxygen, O_b_ = bridging oxygen, and O_c_ = chain oxygen. M_s_ = substitutional metal heteroatom and M_i_ = interstitial metal heteroatom. (b) Photographs showing V_2_O_5_ dispersion color change as a function of SnCl_2_ concentration and reaction time at 65 °C in an inert N_2_ environment.

α-V_2_O_5_ is thermodynamically favored to crystallize into the orthorhombic (*Pmmn* symmetry) structure composed of VO_5_ pyramids (Figure 1a).^32^ Oxygen atoms exist in three distinct bonding states: Chain oxygens (O_c_) are bonded to two V^5+^ atoms, bridging oxygen (O_b_) is bonded to three V^5+^ atoms, and the terminal vanadyl oxygen (O_t_) is bonded to a single V^5+^ atom and protrudes into the quasi-van der Waals (vdW) gap. Compared to the double and triple coordination of chain and bridging oxygens, respectively, the terminal oxygen has the lowest energy reaction pathway to form vacancies that are known to modulate the V_2_O_5_ Fermi level and conductivity.^33^ Metal heteroatoms can incorporate into the V_2_O_5_ lattice substitutionally (*M*_s_) for high valency metals (≥3+) or interstitially (*M*_i_) for lower valency (<3+).^34^ Interstitial heteroatoms typically sit in the quasi-van der Waals gap but alternative sites have been demonstrated.^35^ Interstitial sites can be readily accessed through shear distortions that alter the stacking sequences of infinite V_2_O_5_ sheets.^2,3^

The addition of SnCl_2_ to suspensions of V_2_O_5_ nanoparticles at concentrations ranging from 1 to 100 mM produced visual (and tunable) color change, even at room temperature. The reaction proceeded more rapidly at 65 °C to produce colors evolving from yellow to army green to near black over the course of 3 h (Figure 1b). Treating metal oxides with a solution of SnCl_2_ has been shown to significantly affect the optical and electrical behavior through a presumed intercalation reaction.^36,37^ Since SnCl_2_ is known to disproportionate (2Sn^2+^Cl_2_ → Sn^0^ + Sn^4+^Cl_4_) in the presence of acetone and tartaric acid,^37,38^ several publications have proposed that zerovalent *Sn*^0^ intercalates into the van der Waals gap of metal oxides and other layered chalcogenide hosts due to reaction with solutions of SnCl_2_.^39^ However, we observe the same color change with and without tartaric acid in a variety of solvents (Methods), suggesting this Sn^0^ mechanism is unlikely.

**Figure 2:**
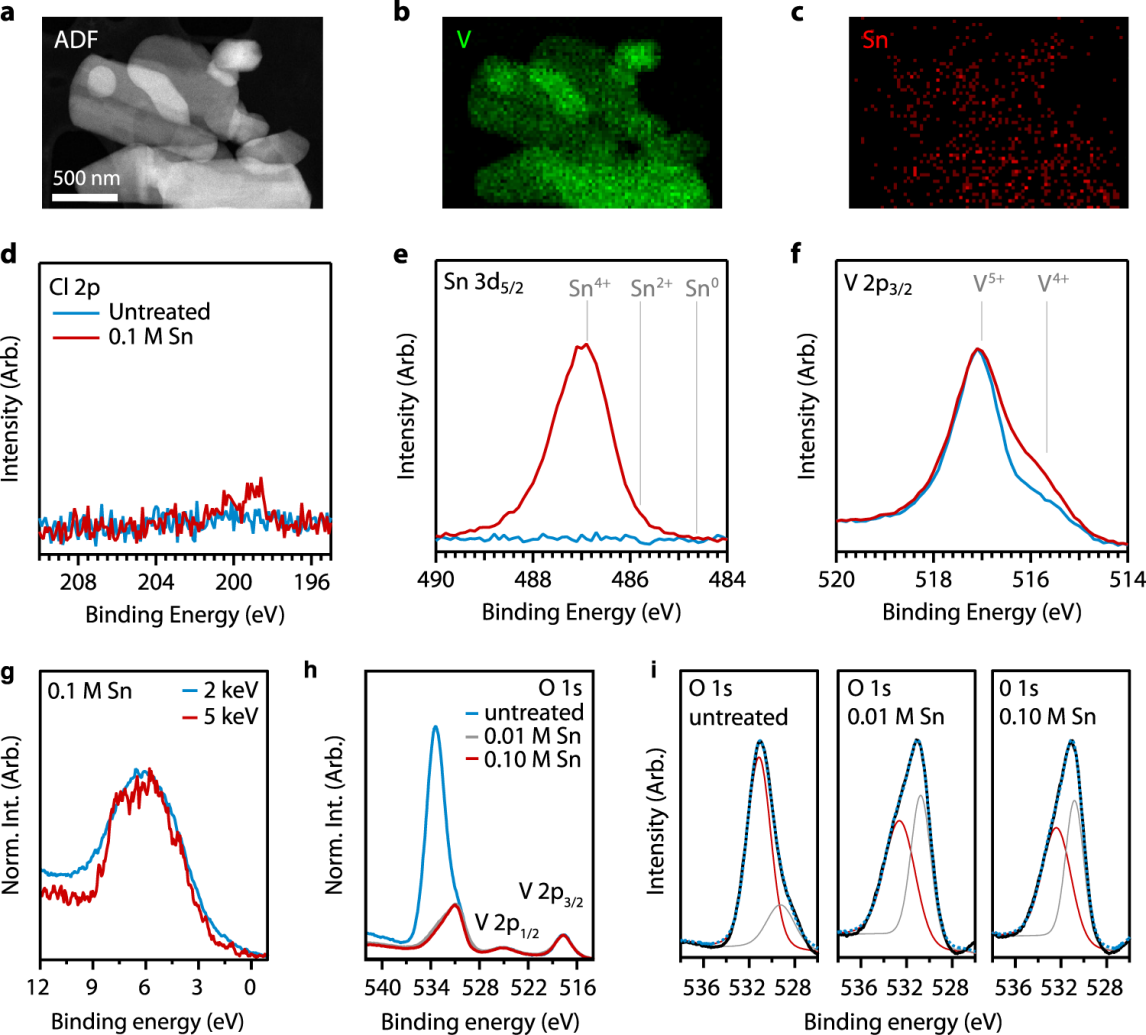
Composition of SnCl_2_-treated V_2_O_5_. (a) Annular dark-field (ADF) STEM image of SnCl_2_-treated V_2_O_5_. (b, c) Energy-dispersive X-ray map of vanadium (b) and tin (c) over the same area as the ADF image. (d–f) XPS core-level spectra of V_2_O_5_ and 0.1 M SnCl_2_-treated V_2_O_5_ highlighting the (d) Cl 2p, (e) Sn 3d_5/2_, and (f) V 2p_3/2_ regions. (g) Energy-variant valence band HAXPES spectra of 0.10 M SnCl_2_-treated V_2_O_5_ are closely overlapped at 2 and 5 keV excitation, which suggests the absence of 5s^2^ stereochemically active lone pairs characteristic of divalent tin. This confirms the nominally tetravalent oxidation state assignment of the incorporated Sn. (h) V 2p and O 1s core-level HAXPES spectra acquired at 2 keV normalized to V 2p_3/2_ depict a clear decrease in the relative O 1s intensity of the SnCl_2_-treated compounds, which is attributed to the formation of oxygen vacancies. (i) Change in the O 1s peak shape and fits illustrate the evolution of the oxygen local environment because of the oxygen vacancies and tin incorporation. The dashed blue line is a composite fit.

### Vanadium Reduction and Tin Doping

Redox chemistry is a more likely result than Sn^0^ intercalation when SnCl_2_ solutions are combined with V_2_O_5_ nanoparticles. SnCl_2_ is known to be a strong reducing agent, and V_2_O_5_ is conversely a strong oxidizing agent, which is used industrially in the contact process to produce sulfuric acid. It has also been shown to undergo phase transitions, e.g., to VO_2_, under cathodic bias by losing oxygen.^40^ The standard redox potential of the Sn^2+^/Sn^4+^ couple is +0.15 V, while that of the V^4+^/V^5+^ couple is +1.0 V (both vs NHE), providing a ca. 0.85 V driving force for the redox reaction in which electrons are transferred from SnCl_2_ to the α-V_2_O_5_ lattice, oxidizing Sn^2+^ to Sn^4+^. The electrons liberated from Sn^2+^ should thus reduce V^5+^ to V^4+^, which must be balanced by the loss of an O atom from the α-V_2_O_5_ lattice. Sn^4+^ has also been shown to act as a substitutional dopant in α-V_2_O_5_.^8^

Based on the above considerations, we propose the following redox reaction that accommodates the possibility of Sn substitution:
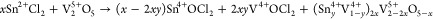
1where *x* is the fraction of oxygen vacancies and *y* is the fraction of Sn^4+^ that exchanges with V^4+^ lattice sites in the mixed-valence vanadium oxide. As the reaction proceeds, the ratio of V^4+^ and Sn^4+^ to V^5+^ increases in vanadium oxide. To remove one oxygen atom to form a vacancy, two vanadium atoms must be reduced or a vanadium atom must be replaced by Sn^4+^. In this scheme, oxygen atoms are abstracted from the V_2_O_5_ lattice to form the solvated SnOCl_2_ or VOCl_2_. Many solvates of VOCl_2_ are known,^41^ and SnCl_2_ is known to be oxidized by molecular oxygen and should readily react with an oxygen radical abstracted from the V_2_O_5_ lattice.^42^ Messin and Janier-Dubry showed that the reaction product was tin oxychloride rather than SnO_2_ and Cl_2_,^43^ and the reaction only proceeded in coordinating solvents, including tetrahydrofuran, pyridine, and acetonitrile. If *y* = 0 and the reaction occurs to yield thermodynamically stable crystal structures, each of the known Wadsley phases of mixed-valence vanadium oxides are possible as *x* is increased: V_3_O_7_ at *x* = 1/3, V_4_O_9_ at *x* = 1/2, V_6_O_13_ at *x* = 2/3, and VO_2_ at *x* = 1. Density functional theory (DFT) studies have predicted midgap states due to both oxygen vacancies (change in *x*)^44,45^ and tin incorporation (change in *y*),^46^ which is consistent with our observation in the change in color of V_2_O_5_.

We confirm that Sn is incorporated into the vanadium oxide nanoparticles through energy-dispersive X-ray spectroscopy (EDX) and X-ray photoelectron spectroscopy (XPS) analyses, including hard X-ray photoemission spectroscopy (HAXPES) probing tens of nanometers into the bulk (Figure 2). Annular dark-field (ADF) scanning transmission electron microscopy (STEM) images show that the SnCl_2_-treated α-V_2_O_5_ nanoparticles are polydisperse with nonspherical shapes and typical dimensions between 100 and 500 nm (Figure 2a). EDX maps of the same V_2_O_5_ nanoparticles show that V (Figure 2b) and Sn (Figure 2c) are uniformly distributed in the V_2_O_5_ nanoparticles and point to the absence of Sn or Sn-rich phases segregated to the nanoparticle surface. XPS allows us to confirm that the Sn detected in EDX is not due to residual SnCl_2_ precursor, as the Cl 2p signal is negligible (Figure 2d) compared to the strong signal exhibited by the Sn 3d_5/2_ peak (Figure 2e). We employed a thorough washing procedure of repeated centrifugation and dispersion in methanol that effectively removes all Cl byproducts (Methods).

XPS reveals the presence of surface (top ∼10 nm) Sn and oxygen vacancies in the vanadium oxide lattice. The oxidation state of tin in the vanadium oxide samples is Sn^4+^ rather than Sn^2+^ or Sn^0^, which is consistent with eq 1 (Figure 2e). Based on SnO_2_/SnO studies, Sn^4+^ exists in an octahedral bonding environment with six oxygens and is expected to have a 0.7 eV higher binding energy (ca. 486.4 eV) than Sn^2+^ (ca. 485.7).^47^ Sn^0^ is expected at even lower binding energies (ca. 484.5 eV).^48^ V^4+^ (ca. 516 eV) and V^5+^ (ca. 517 eV) have an electron binding energy difference of roughly 1 eV (Figure 2f).^49,50^ The presence of V^4+^ is an indication of oxygen vacancies, as two V^5+^ atoms must be reduced to V^4+^ or replaced by Sn^4+^ to form a single oxygen vacancy (eq 1).

As topochemical reactions can yield transformations confined to the surface, HAXPES was utilized to probe Sn substitution and O vacancy formation within the bulk material. The higher incident energies of high-resolution synchrotron HAXPES yield increased penetration depths (∼10–30 nm) compared to laboratory XPS sources.^51^ HAXPES core-level spectra were first measured at an excitation energy of 2 keV, exhibiting features centered at ca. 496.6 eV and ca. 488.2 eV. These correspond to Sn^4+^ 3d_3/2_ and 3d_5/2_, which suggests that the Sn^4+^ substitution extends further into the bulk from the surface than can be probed with laboratory XPS (Figure S1). The intensities of the Sn^4+^ 3d_3/2_ and 3d_5/2_ peaks also increase upon treatment of V_2_O_5_ with higher concentrations of SnCl_2_, suggesting increased substitution of Sn^4+^. Core-level HAXPES was also unable to detect Cl within the bulk of the material (Figure S2).

The Sn oxidation state was further confirmed as nominally tetravalent by measuring the valence band using energy-variant HAXPES. Sn^2+^ manifests the stereochemical activity of its 5s^2^ electron lone pairs upon appropriate anion hybridization, whereas Sn^4+^ does not.^52^ The presence of stereochemically active lone pairs can be directly observed through energy-variant HAXPES since the photoionization cross section of the filled 5s/6s orbitals of p-block cations do not decay as sharply at high incident photon energies.^19^ Therefore, the valence band HAXPES spectra collected at higher incident energies would more prominently feature orbital contributions from the Sn^2+^ lone pair of electrons. Previous literature has shown that a distinct feature in the energy-variant valence band HAXPES spectrum of β-Sn_0.33_V_2_O_5_ arises at ca. 11 eV and increases in intensity upon increasing photon energy, indicative of the stereochemical activity of the Sn^2+^ lone pair of electrons.^53^ However, the energy-variant valence band HAXPES spectra of 0.10 M SnCl_2_-treated V_2_O_5_ collected at 2 and 5 keV are virtually identical in terms of shape and intensity (Figure 2g). This result can only be obtained if the Sn atoms do not contain stereochemically active lone pairs, which further supports the assignment of the Sn^4+^ oxidation state.

Normalizing the O 1s and V 2p HAXPES spectra to the V 2p_3/2_ signal clearly depicts a decrease in the O 1s signal upon treatment with SnCl_2_, arising from the presence of oxygen vacancies (Figure 2h). Furthermore, fitting the O 1s peak of the HAXPES spectra allows for the local coordination of the oxygen atoms to be probed in extensive detail. The local oxygen coordination environment clearly evolves upon Sn^4+^ substitution and the formation of lattice oxygen vacancies, as evidenced by the drastic change in the O 1s peak shape (Figure 2i).

**Figure 3:**
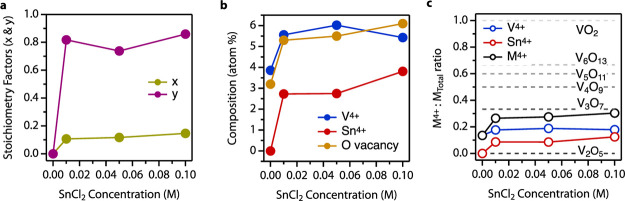
(a) Evolution of *x* (oxygen vacancies) and *y* (Sn^4+^ substitution) from eq 1 as a function of SnCl_2_ concentration for a 90 min reaction as determined from XPS (Methods). (b, c) Composition of V^4+^ and Sn^4+^ (b) and ratio of M^4+^ to total metals in the sample (c) as a function of SnCl_2_ concentration for a 90 min reaction as determined from XPS. Dashed lines correspond to expected M^4+^:M_Total_ ratio for known thermodynamically stable vanadium oxide compounds.

Varying the SnCl_2_ concentration allows us to systematically tune the concentration of oxygen vacancies and Sn substitution in the vanadium oxide host. For 90 min reactions employing SnCl_2_ concentrations from 0.0 to 0.1 M SnCl_2_, oxygen vacancy concentration (*x*) monotonically increases, from 0.10 to 0.15 with increasing SnCl_2_ concentration, based on XPS (Figure 3a). In the same reaction series, the ratio of Sn^4+^ to V^4+^ (*y*, eq 1) increases from 0 to ∼0.8 and remains relatively constant with increasing SnCl_2_. The data suggest that Sn substitution favors the formation of V^4+^. We also present the data in absolute atomic percent (Figure 3b). There is a ∼4 atom % population of V^4+^ in the untreated α-V_2_O_5_, which increases to >6 atom % after treatment with 0.1 M SnCl_2_. Sn^4+^ monotonically increases up to >3.5 atom % after exposure to 0.1 M SnCl_2_ for 90 min. The result of V^4+^ formation and Sn^4+^ incorporation leads to a significant loss in oxygen in the lattice, with oxygen vacancies reaching ∼6% after treating the sample with 0.1 M SnCl_2_ for 90 min (Figure 3b).

### Topochemical Transformation

In prior work, restructuring was not observed in nanoparticles with up to 5% oxygen vacancy concentration.^33^ Here, we show a vacancy concentration of up to ∼6% based on XPS tin and vanadium peaks; HAXPES results indicate that oxygen vacancy formation is not just on the surface but extends at least 30 nm into the bulk. The relative concentration of 4+ metal atoms (M^4+^ = Sn^4+^ + V^4+^) to the total number of atoms in the lattice approaches the concentration expected for V_3_O_7_ (Figure 3c). We thus expect additional phases of vanadium oxide to appear. High-temperature reduction of stoichiometric α-V_2_O_5_ typically proceeds through multiple simultaneous Wadsley-phase intermediates and ends with V_2_O_3_, a Magnéli phase.^17^ High-temperature reduction produces thermodynamically stable intermediate crystal structures, which are accessed through a series of relatively low-energy crystallographic shear transformations along different directions that eliminate ordered arrays of oxygen vacancies, whereas our topochemical approach yields a crystal structure that preserves long-range ordering.

**Figure 4:**
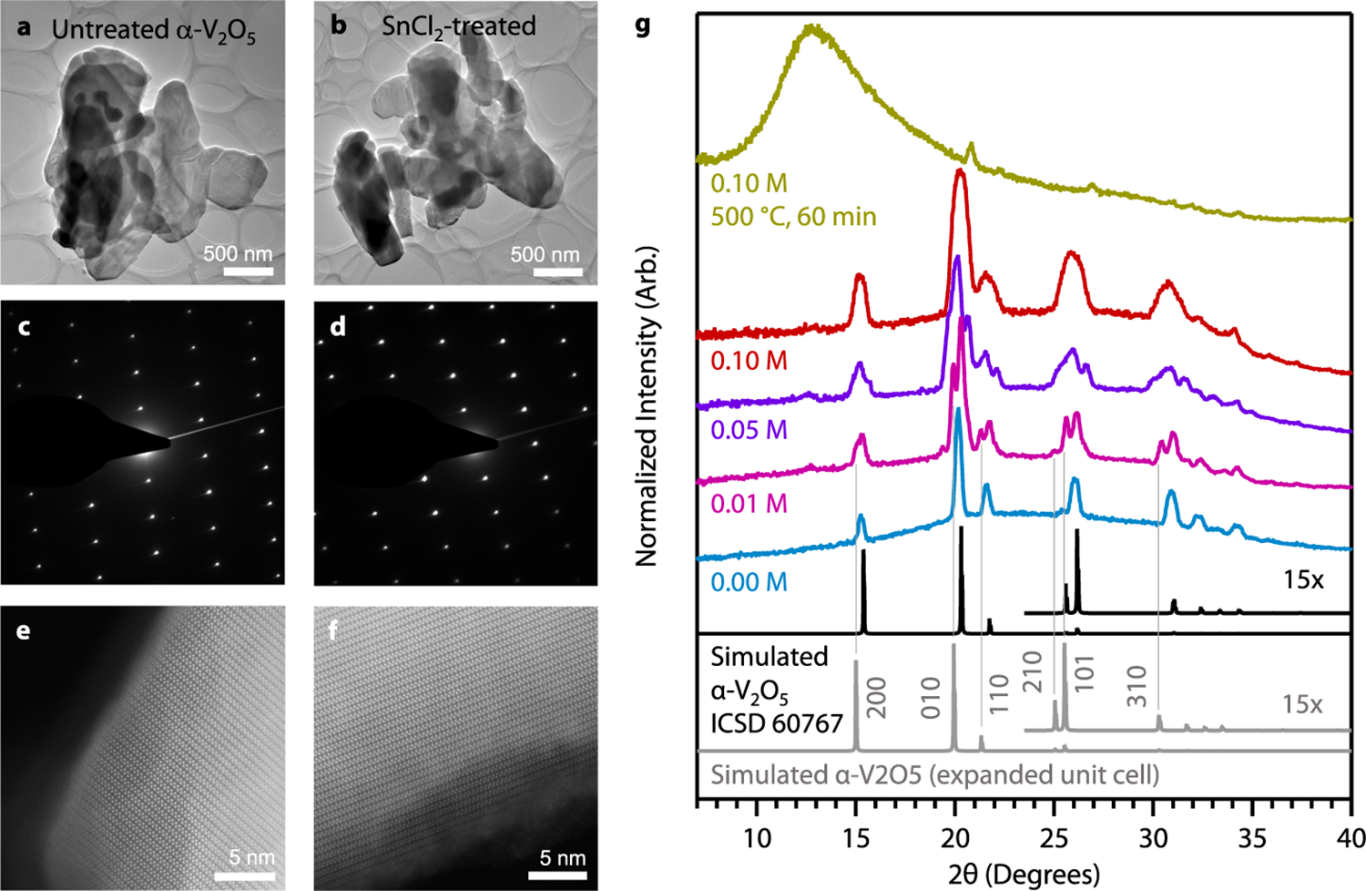
Structural evolution of V_2_O_5_ from SnCl_2_ redox chemistry. (a, b) Transmission electron microscopic (TEM) images of untreated α-V_2_O_5_ (a) and 0.05 M SnCl_2_-treated V_2_O_5_ (b). (c, d) Selected-area diffraction images of untreated α-V_2_O_5_ (c) and 0.05 M SnCl_2_-treated V_2_O_5_ (d). (e, f) Annular dark-field scanning transmission electron microscopic (ADF-STEM) images of untreated α-V_2_O_5_ (e) and 0.05 M SnCl_2_-treated V_2_O_5_ (f). (g) Powder X-ray diffraction patterns of untreated α-V_2_O_5_ (0.00 M) and V_2_O_5_ samples with increasing SnCl_2_ concentration. The 2θ axis is relative to Cu Kα (1.5406 Å, 8.04 eV) radiation. The α-V_2_O_5_ pattern was simulated and expanded using VESTA software from ICSD 60767.

Transmission electron microscopic (TEM) images show that untreated α-V_2_O_5_ nanoparticles are polydisperse and remain unchanged after treatment with SnCl_2_ (Figure 4a,b). Selected-area diffraction patterns (Figure 4c,d) show that the transformations induced by SnCl_2_ were topotactic in nature, despite the large changes in stoichiometry. The materials maintain their crystallinity with similar electron diffraction patterns, which suggests that the overall framework is preserved. ADF-STEM images of the edge of V_2_O_5_ nanoparticles before and after SnCl_2_ treatment show lattice fringes extending to within ∼1 nm of the particle surface with minimal changes in lattice spacing (Figure 4e,f). Noncrystalline material at the surface may be amorphous vanadium oxide or simply adventitious carbon.

X-ray diffraction (XRD) confirmed that the vanadium oxide samples maintain their crystal symmetry after topochemical reduction and tin incorporation. Untreated α-V_2_O_5_ exhibited the expected orthorhombic reflections indicative of *Pmmn* (No. 59) symmetry (Figure 4g).^54^ After SnCl_2_ treatment, new reflections emerged that we attribute to an expansion of the unit cell (Figure 4d), but not the appearance of new crystalline phases, oxychlorides, or tin oxides. Previous studies on substitutional tin doping report inconsistent lattice parameters based on powder X-ray diffraction, with all works reporting expansion of the *a*-axis and either expansion or contraction of the *b* and *c* axes.^7−10^ Studies on the analogous α-MoO_3_ system also showed that increasing oxygen vacancy content led to an increase in unit cell dimensions.^55^ A 90 min treatment with 0.01 M SnCl_2_ yields a significantly expanded α-V_2_O_5_ unit cell with shifts to lower angles for the prominent 010, 110, 210, and 310 reflections.

The evolution of the XRD patterns resembles reports of hydrogen intercalation into α-V_2_O_5_ where reflections shift, split, and evolve with increasing hydrogen concentration but without signs of additional phases.^56^ At 0.1 M SnCl_2_, the distinct reflections merge into broad peaks, indicating short-range disorder but a crystal that has still maintained a long-range order. Raman spectroscopy on the same samples also indicated no new phases and maintained bonding motifs within the lattice (Figure S5). We did not observe multiple peaks for each reflection using electron diffraction (Figure 4d), but the pattern contracted after treatment, which is consistent with the expanded lattice observed in XRD (Figure S4). Since powder X-ray diffraction is volume-weighted over a large area compared to electron diffraction of individual particles in the electron microscope, we hypothesize that the appearance of multiple peaks at each reflection in XRD is due to a sampling of multiple nanoparticle populations where smaller particles react faster than larger ones. We observe a similar XRD pattern evolution when using acetone as the solvent (Figure S6).

Samples that were treated with 0.1 M SnCl_2_ and subsequently annealed in air at 500 °C became mostly amorphous, indicating that the high oxygen vacancy concentration and substitutional Sn achieved here are only kinetically accessible through low-temperature topochemistry (Figure 4g). The preservation of the average structure with a layered orthorhombic leitmotif, evidenced in lattice-resolved TEM imaging, electron diffraction, and powder X-ray diffraction, indicates that crystallographic shear transformation is considerably impeded. Shear transformations are characteristic of binary vanadium oxides and drive structural transformations through a sequence of phases. We posit that substitutional Sn atoms positioned on the vanadium sublattice can prevent vacancy reordering and crystallographic shear transformations in a manner reminiscent of dopants freezing motion of dislocations through periodic lattices.^57^

Topochemical transformations are known to occur in α-V_2_O_5_, but exchange at the vanadium site for other cations has only been shown for bottom-up synthesis. We compiled known V_2_O_5_-derived materials with heteroatoms incorporated into the structure that maintain the *Pmmn* space group symmetry and compared them to the V_2_O_5_ sample treated with 0.01 M SnCl_2_ (Table 1). Materials are grouped by the location of the heteroatoms. The most heavily studied subset of these materials is for battery cathode applications and can be produced topochemically or electrochemically by intercalation of alkali or alkaline earth metals (e.g., LiV_2_O_5_,^58^ NaV_2_O_5_,^59^ and CaV_2_O_5_^60^). In these materials, the intercalated ion has a valency ≤2 and a large crystal radius of ≥0.9 Å.^62^ The atoms insert interstitially into the vdW gap where they are charge-balanced by reduced vanadium sites (V^4+^) that maintain their 5-fold oxygen coordination. vdW gap insertion leads to a large change in the *b*-axis of the unit cell compared to the parent compound (Δ*b* > 6%). The relative amount of Sn^4+^ to V^4+^ based on XPS and HAXPES makes interstitial incorporation of Sn^4+^ into V_2_O_5_ unlikely. Four V^4+^ atoms are needed to balance a single interstitial Sn^4+^ atom. XPS shows that the V^4+^-to-Sn^4+^ ratio is closer to 2 (Figure 3b).

**Table 1:**
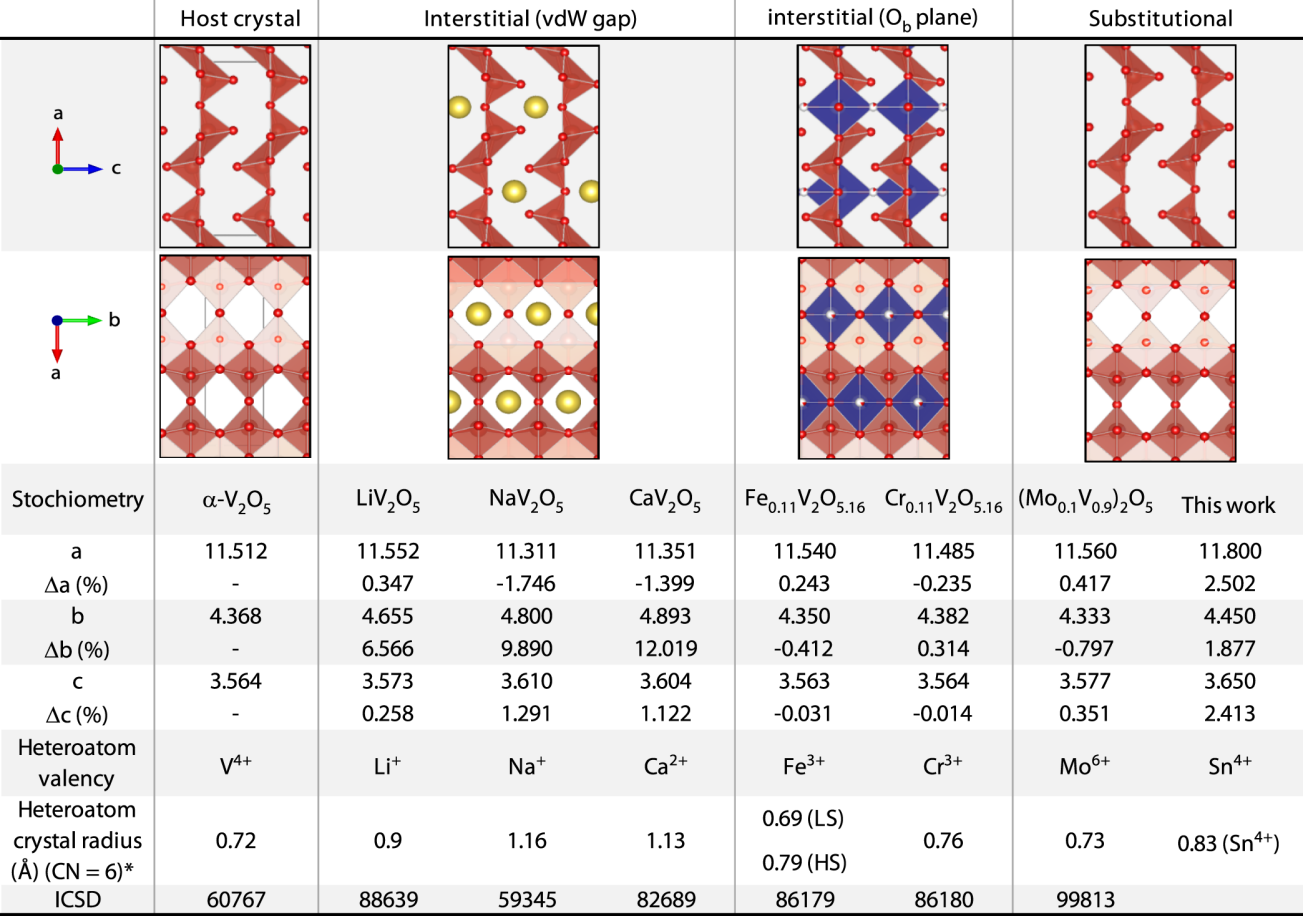
Characteristics of V_2_O_5_ Compounds Exhibiting Orthorhombic *Pmmn* Space Group Symmetry Broken Down into Three Types of Heteroatom Incorporation^a^

a Large red atoms = vanadium, small red atoms = oxygen, yellow atoms = sodium, and blue octahedra = [CrO_6_]^9–^. Crystal structure characteristics are based on ICSD entries from various literature sources.^14,32,58−61^ vdW = van der Waals. *Crystal radii are based on Shannon,^62^ with the exception of Sn^2+^, which is based on Sidey et al.^63^ LS = low spin, HS = high spin, CN = coordination number.

Higher valency ions such as Cr^3+^ and Fe^3+^ have smaller crystal radii and have been shown to insert interstitially close to the plane made by the bridging oxygens rather than within the vdW gap. Oxygen atoms also accompany the interstitial cations to increase the overall V:O ratio, in contrast to what we observe here. Cr_0.11_V_2_O_5.16_ and Fe_0.11_V_2_O_5.16_ have been synthesized from a bottom-up sol-gel process, but there are no reports of topochemical access of these compounds.^61^

The radius of Sn^4+^ (0.83 Å) in 6-fold coordination is larger than that of V^4+^ (0.72 Å), but not as large as ions known to intercalate into the vdW gap. DFT shows that high valency metals (>3+) such as Sn^4+^ are more thermodynamically favored as substitutional rather than interstitial dopants.^34^ Though Sn^4+^, Ti^4+^, and Bi^3+^ have been reported as substitutional dopants in the V_2_O_5_ lattice,^8,11,12^ only Mo^6+^-doped V_2_O_5_ had a single-crystal or high-resolution structure data available.^14,16^ Mo^6+^ has nearly the same crystal radius as V^5+^, whereas Sn^4+^ is ∼13% larger, which leads to larger structural distortions in each direction of the unit cell. Delocalization of electron density between Mo and V sites was observed in Mo and V K-edge XANES analyses of substitutionally doped V_2_O_5_ indicating a formal Mo valence between +5 and +6.^16^ Notably, substitutional Mo doping considerably expands phase stability regimes by introducing local distortions, such as increased puckering of the V_2_O_5_ layers and canting of the vanadyl oxygens. The suppression of crystallographic shear transformations in the Sn-doped V_2_O_5_ presented here appears to be directly analogous to the suppression of the intercalation-driven layer shearing in Mo-alloyed V_2_O_5_.^16,64^

### Optical and Electrical Properties

**Figure 5:**
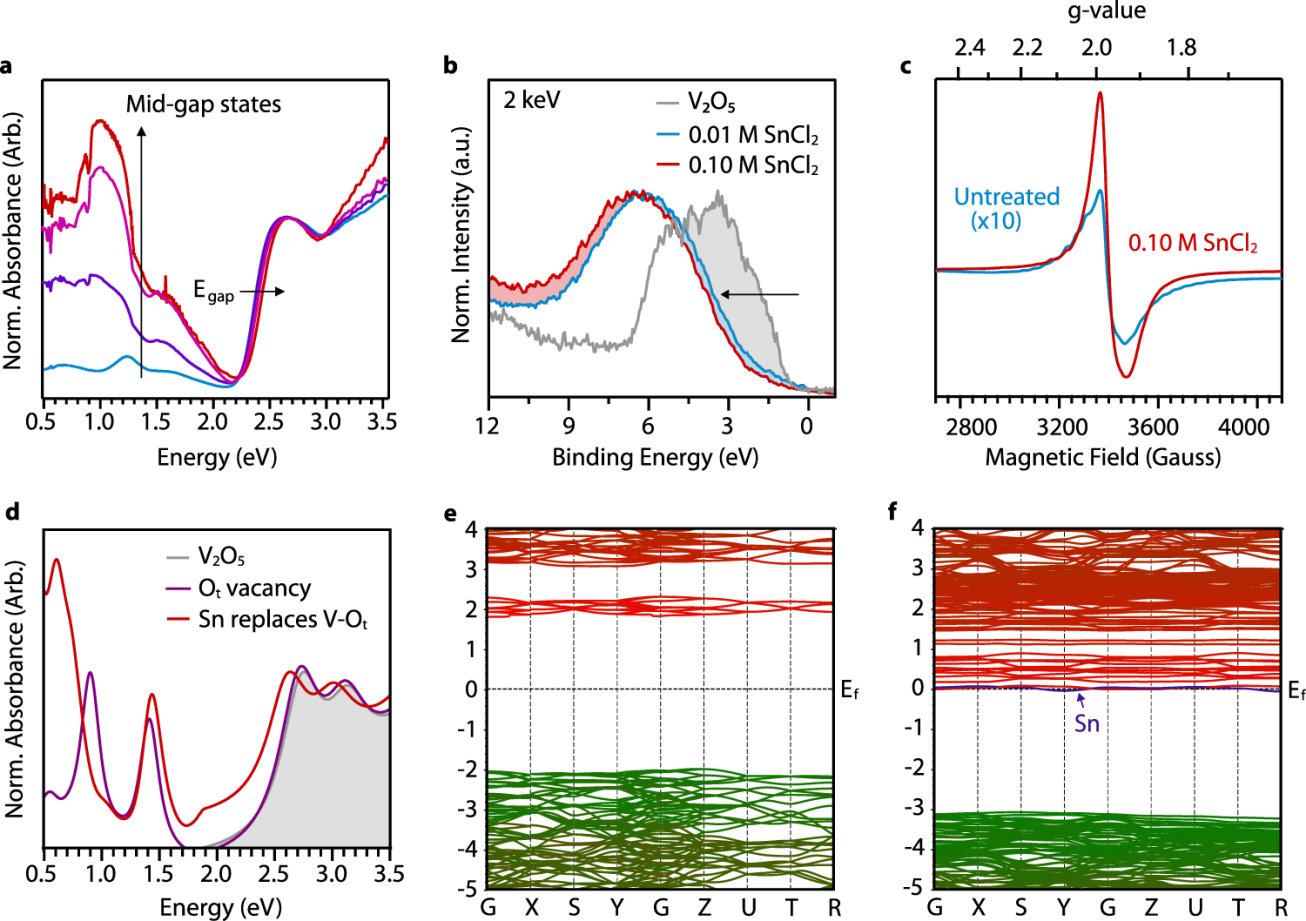
Optical and electrical properties of SnCl_2_-treated V_2_O_5_ nanoparticles. (a) UV–vis–NIR absorbance of α-V_2_O_5_ films as a function of SnCl_2_ treatment. Spectra are normalized at 2.75 eV and offset to account for scattering. (b) Valence band HAXPES spectra of α-V_2_O_5_ and 0.01 and 0.10 M SnCl_2_-treated α-V_2_O_5_ measured at 2 keV show a clear increase in the energy separation between the Fermi level and the valence band edge, consistent with the Moss–Burstein effect arising from a population of conduction band electrons that are thermally excited from midgap vacancy states. (c) Electron paramagnetic resonance (EPR) spectra of untreated α-V_2_O_5_ and 0.1 M SnCl_2_-treated α-V_2_O_5_ collected at 60 K. (d) Absorbance spectra from theoretical calculations based on a quasiparticle self-consistent GW (QSGW) approximation, illustrating midgap absorption bands due to oxygen vacancies and Sn substitution. (e, f) Electronic band structures for undoped (stoichiometric) α-V_2_O_5_ (e) and Sn substitution for V = O_t_ into the α-V_2_O_5_ lattice (f).

The topochemical approach shown here is unique in the V_2_O_5_ literature in installing a p-block cation directly on the vanadium sublattice and demonstrates a new avenue to tailor the properties of the material. Material structure and composition are intimately linked to optical and electronic properties. UV–vis absorption spectroscopy shows that treatment with increasing SnCl_2_ concentration induces a systematic hypsochromic (blue) shift of the primary absorption onset at ca. 2.2 eV, along with the growth of a broad envelope of lower energy optical transitions below 2.2 eV (Figure 5a).

These observations are consistent with the introduction of oxygen vacancies and conduction band electrons. According to previous DFT calculations, we expect oxygen vacancies to give rise to midgap states with energies ranging from ca. 0.69 to 0.95 eV above the valence band maximum. Peaks arise due to corresponding oxygen vacancies in terminal, bridging, and chain coordination geometries/environments.^45^ These states are present even before SnCl_2_ treatment, which is consistent with the observation of V^4+^ in XPS spectra of untreated V_2_O_5_ (Figure 2f). Since these vacancy sites are occupied with electrons,^45^ they give rise to the observed optical transitions that correspond to promotion of these localized electrons to the conduction band, where the sub-band gap peak intensities are proportional to the vacancy density.

The density of occupied vacancy sites is also expected to move the Fermi level (*E*_F_) closer to (or into) the conduction band due to thermal excitation of electrons to the conduction band. The systematic blue shift of the ca. 2.2 eV band gap absorption onset with increasing SnCl_2_ is consistent with the Moss–Burstein shift arising from a progressively increasing *E*_F_, consistent with that observed previously for lithium-modulated vacancy formation in V_2_O_5_.^44^ The position of *E*_F_ was probed with HAXPES, and the valence band of untreated V_2_O_5_ measured at 2 keV is shown in Figure 5b and compared with similar data for 0.01 and 0.10 M SnCl_2_-treated V_2_O_5_. The distance between the valence band edge and *E*_F_ increases upon SnCl_2_ treatment, consistent with the Moss–Burstein shift and the notion that oxygen vacancies contribute electron density to the V_2_O_5_ conduction band. The valence bands measured at 5 keV, which probe up to 30 nm within the bulk material due to the higher incident photon energy, also show a similar trend (Figure S3).

The new sub-band gap states observed in optical absorption from formation of oxygen vacancies in the host lattice led to an increased number of free electrons and V^4+^ (eq 1). Electron paramagnetic resonance (EPR) is sensitive to unpaired electrons and has also been shown to be a useful tool for identifying V^4+^ in vanadium oxides.^65^ The weak EPR signal at g-value ∼2 for the untreated α-V_2_O_5_ that is a superimposed hyperfine structure that indicates coupling of the electron spins to V^4+^ nuclei (Figure 5d). This is consistent with electron doping due to adventitious oxygen vacancies.^66^ Treatment with 0.1 M SnCl_2_ induces a resonance feature that is >10× more intense than the untreated sample, indicating a significant increase in the concentration of free electrons and V^4+^ (Figure 2e). Sn^4+^ and Sn^2+^ centers are not EPR-active and will not contribute to the observed EPR signal.

We verify our experimental findings with theoretical calculations based on quasiparticle self-consistent GW (QSGW) approximation^67^ using the primitive cell of bulk α-V_2_O_5_, which contains 14 atoms. We have reported the electronic properties and optical spectra of bulk α-V_2_O_5_ in our previous work and show the optical spectrum in Figure 5d, where a thorough analysis indicates that the features located at energies above ca. 2.2 eV are purely excitonic in nature.^68^ To simulate the effects of oxygen vacancies and substitutional Sn^4+^ on the electronic structure and optical properties, we constructed an 84-atom supercell. GW is a theory for many-body scattering, and the optical spectra are computed with the full dynamical self-energy, i.e., considering both the electronic eigenvalues and their lifetime effects via the imaginary part of the dynamical self-energy. This allows simulation of several broad midgap (0.5–2.0 eV) optical absorptions when oxygen vacancies and Sn^4+^ are introduced into the lattice (Figure 5d), consistent with measured optical spectra (Figure 5a).

Band structure calculations that complement optical absorptions show a 3.8 eV band gap (and 2.2 eV optical gap) and Fermi level (*E*_F_) in the middle of the band gap, as expected for undoped V_2_O_5_ (Figure 5e). Simulations reveal that formation of a vanadyl oxygen (O_t_) vacancy adds two electrons, raising the Fermi level to the conduction band minimum. Conversely, it is pushed into the valence hole-like states when Sn^4+^ dopes the system. When one Sn^4+^ atom replaces a V–O_t_ unit, it compensates for the oxygen vacancy and the system effectively gains one net electron. The overall electronic structure is metallic with the partially filled V (red) and Sn (blue) states crossing the Fermi level (Figure 5f).

### Thin Film Chemistry and Devices

**Figure 6:**
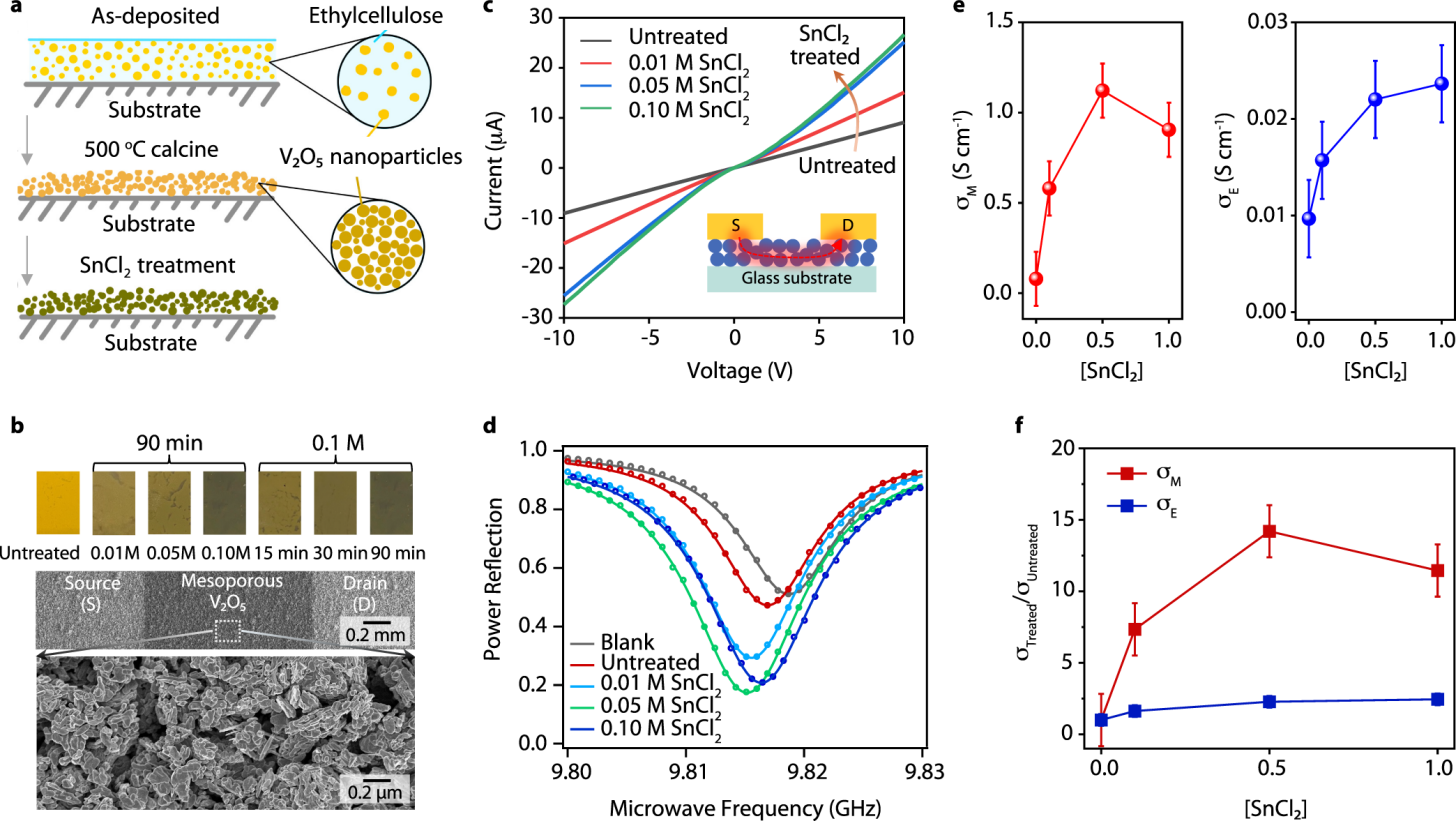
Optical and electrical characterization of the SnCl_2_-treated V_2_O_5_ film. (a) Cartoon illustrating preparation and treatment of mesoporous α-V_2_O_5_ films. (b) Photographs of mesoporous films treated with increasing concentration of SnCl_2_ for 90 min and evolution of a treatment with 0.1 M SnCl_2_ as a function of time (top panel). The bottom panel presents a scanning electron microscopic (SEM) image of the Au/α-V_2_O_5_/Au device, highlighting the source/drain contact on the mesoporous α-V_2_O_5_ film, and a zoomed-in view of the mesoporous α-V_2_O_5_ structure within the channel. (c) Current–voltage characteristics of the Au/α-V_2_O_5_/Au device corresponding to various concentrations of SnCl_2_ treatment: 0.01, 0.05, and 0.10 M, each treated for 90 min. The inset shows a schematic side view of the device, illustrating the current flow between the source and drain contacts through the mesoporous α-V_2_O_5_ film. (d) Microwave conductivity power reflection resonance curves for untreated and SnCl_2_-treated films at various concentrations (0.01, 0.05, and 0.10 M). Circles represent the experimental data, while lines indicate the fitted data. The blank refers to the quartz substrate prior to the deposition of the mesoporous α-V_2_O_5_ film. (e) Conductivity values extracted from noncontact microwave conductivity and two-terminal device electrical measurements for the film treated with different concentrations of SnCl_2_. (f) Comparison of the conductivity ratio between untreated and SnCl_2_-treated sample, as measured by noncontact microwave conductivity and for two-terminal devices.

We demonstrate that topochemical SnCl_2_ reduction chemistry is also possible in mesoporous thin films of α-V_2_O_5_ fabricated by formulating a viscous ethylcellulose-based ink that may be processed to deposit films by using common solution deposition techniques. The inks are analogous to the TiO_2_ nanoparticle slurries used to form electrodes commonly employed in dye-sensitized and perovskite solar cells.^69^ Here, we leverage either bar coating or spin coating to control the film thickness and morphology (Methods). Calcination at 500 °C in air burns off the ethylcellulose matrix and brings the α-V_2_O_5_ nanoparticles into intimate contact with one another to improve charge transport. Areas where ethylcellulose existed in the film are transformed into voids to yield a mesoporous film (Figure 6a). Calcination also oxidizes V_2_O_5_ with molecular oxygen to fill some oxygen vacancies that exist in as-deposited α-V_2_O_5_. SnCl_2_ treatment resulted in the same color changes observed in the colloidal dispersions of bulk powders (Figure 1b) with increasing SnCl_2_ concentration or treatment time, producing darker green films, which indicates that the chemistry is transferable to thin films (Figure 6b, top).

Electrical transport within the mesoporous vanadium oxide film was characterized in two-terminal planar devices. These devices comprise gold (Au) contacts, which were thermally evaporated onto the α-V_2_O_5_ film to form the source/drain electrodes. SEM images of the devices offer a closer examination of the mesoporous α-V_2_O_5_ film’s microstructure within the active channel of the device (Figure 6b, bottom). Despite the high porosity of the film, the individual particles are sufficiently connected to allow for a percolation network between the source and drain electrodes (Figure 6c, inset). Current–voltage (*I*–*V*) characteristics of the two-terminal devices demonstrate that film conductivity (the slope of the *I*–*V* curve) is improved by increasing the SnCl_2_ concentration (Figure 6c). The *I*–*V* characteristics of these Au/V_2_O_5_/Au devices exhibit nonlinear, symmetric behavior, expected for a metal/semiconductor/metal junction.

We further investigated the impact of SnCl_2_ treatment on the electronic properties of V_2_O_5_ films through dark microwave conductivity and power reflection measurements. Dark microwave conductivity, a noncontact, high-frequency spectroscopic technique, probes the complex dielectric function of a thin film sample via modulation of the quality factor of a loaded ca. 10 GHz resonant cavity. Details on the analysis of the cavity resonance can be found in recent studies.^70^ The cavity resonance for an untreated α-V_2_O_5_ nanoparticle film is both deeper and shifted to lower resonance frequency than that of the blank quartz substrate on which it was deposited (Figure 6d). The deeper resonance indicates that the untreated sample has appreciable 10 GHz conductance, most likely arising from the native concentration of oxygen vacancies and free electrons, whereas the bathochromic (red) shift of the resonance frequency arises primarily from the increased relative permittivity of α-V_2_O_5_. Increasing SnCl_2_ treatment concentration induces a deepening and broadening of the cavity resonance, along with a further red shift of the resonance frequency, indicating an increase in both the dielectric constant and conductance of the film upon SnCl_2_ treatment. These observations are consistent with an increase in the oxygen vacancy concentration and the incorporation of Sn^4+^ into the α-V_2_O_5_ crystal structure. For the α-V_2_O_5_ film treated with 0.1 M SnCl_2_, the resonance frequency shows a slight blue shift compared to films treated with 0.05 and 0.01 M SnCl_2_. We tentatively attribute this to variations in the thickness of the α-V_2_O_5_ layer with high concentrations of SnCl_2_.

To quantify the changes in conductivity, we derived conductivity values from both microwave conductivity and electrical measurements (Figure 6e,f). These results reveal a clear trend of increasing conductivity with higher SnCl_2_ concentrations, consistent with the rise in the free carrier concentrations inferred from the absorbance and EPR measurements discussed earlier. Notably, microwave conductivity (σ_M_) increases by a factor of ca. 14 over the same concentration range of SnCl_2_ treatments that produces a much smaller ca. 2–3-fold increase in two-terminal DC conductivity (σ_E_) and a ca. 6-fold increase in four-terminal van der Pauw DC conductivity. This discrepancy arises from the different transport length scales probed by the two measurement techniques. Microwave conductivity can probe *local transport* occurring within the ca. 100 ps period of the 10 GHz probe (i.e., down to the single-particle level), whereas the DC conductivity measurement is only sensitive to long-range charge transport that occurs over a millimeter length scale through the percolated network of α-V_2_O_5_ particles and that is likely limited by transport across barriers between particles. It is this difference in the length scales probed by the techniques that results in a 10 GHz conductivity that is >10 times larger than that extracted using the DC conductivity techniques. It is important to note that the initial two-terminal DC measurements can often lead to inflated resistance values due to contributions from contact and lead resistances. In contrast, the van der Pauw method (a four-terminal DC measurement) minimizes errors caused by contact resistance and provides a more accurate reflection of the material’s long-range electrical resistance.

The dark microwave conductivity and DC conductivity measurements both confirm that the topochemical transformation of V_2_O_5_ with SnCl_2_ introduces conductive free electrons. The observed macroscopic device characteristics confirm that the optoelectronic changes induced by SnCl_2_ are not confined to the isolated V_2_O_5_ nanoparticles, and long-range transport is possible through the mesoporous V_2_O_5_ film. The Fermi level, carrier density, and long-range conductivity can be finely tuned for solution-deposited mesoporous films of the V_2_O_5_ nanoparticles by low-temperature, solution-phase topochemical SnCl_2_ reduction chemistry. The simultaneous achievement of good interparticle electronic communication and mesoporous film structure suggests that these films and devices could be good candidates for resistive switching devices based on, e.g., electrochemical random access memory (ECRAM) platforms.^71^

## Conclusions

In summary, we demonstrated topochemical reduction of V^5+^ to V^4+^ and substitutional Sn^4+^ incorporation into α-V_2_O_5_ by utilizing a SnCl_2_ solution at a low temperature of 65 °C. The redox process modifies the composition of α-V_2_O_5_ without changing the parent crystal structure and, by substitutional Sn^4+^ incorporation on the vanadium sublattice, affords a distinctive means of engineering oxygen vacancy and free electron concentrations without engendering transformations through a series of Wadsley phases. The SnCl_2_ treatment is viable at room temperature and can be tuned using the SnCl_2_ concentration and reaction time/temperature, which yield control over optoelectronic properties. Optical spectroscopy identified sub-band gap states associated with oxygen vacancies, and electron paramagnetic resonance spectroscopy and dark microwave conductivity measurement demonstrated an increase in free electrons because of the topochemical transformation. We showed that the SnCl_2_ chemistry can also be applied to mesoporous α-V_2_O_5_ films fabricated via scalable solution deposition techniques. Electrical transport in the mesoporous films was probed with current–voltage analysis of two-terminal (four-terminal) devices and noncontact microwave conductivity measurements. Higher SnCl_2_ concentrations led to a ca. 2.3-fold (6.1-fold) increase in conductivity in two-terminal (four-terminal) devices and ca. 14-fold increase in microwave conductivity measurements, indicating that our SnCl_2_ chemistry may be applied to films, but the mesoporous network limits mobility. Our work serves as a valuable demonstration of tailoring the vanadium–oxygen system through post-synthetic top-down topochemical manipulation to impart electronic functionality as an alternative to bottom-up synthesis of exotic phases.

## Methods

### Chemicals

20 wt % α-V_2_O_5_ dispersed in acetone was purchased from US Research Nanomaterials, Inc. Anhydrous methanol, acetone, and DMSO were purchased from Sigma-Aldrich, transferred into a N_2_-atmosphere glovebox, and stored over activated 3 Å molecular sieves. SnCl_2_ was purchased from Sigma-Aldrich and used as received and stored in a N_2_ glovebox. We note that handling nanomaterials poses health risks and vanadium oxides are often perceived to be carcinogenic, though they are used industrially for chemical synthesis; recent toxicology studies show that the carcinogenic nature of V_2_O_5_ requires further study.^72^ Regardless, appropriate personal protective equipment should be used when handling these materials.

### V_2_O_5_ Ink Preparation and Film Deposition

To fabricate the films for UV–vis–NIR absorption and dark microwave conductivity measurements, the α-V_2_O_5_ ink was deposited onto precut quartz substrates using either bar-coating or spin-coating methods in air. The Quartz/V_2_O_5_ sample underwent annealing in an oven for 30 min at 500 °C in ambient conditions. Optical profilometry measurements to determine the film thickness were performed on a Keyence VHX-7000 3D optical profilometer.

### SnCl_2_ Treatment

In the case of doped samples, α-V_2_O_5_ dispersed in acetone was weighed out in a vial of air. Acetone was evaporated from the sample on a hot plate and transferred as dry α-V_2_O_5_ powder to a N_2_-atmosphere glovebox. SnCl_2_ solution in methanol was added to the V_2_O_5_ powder at varying concentrations and for varying treatment times within the N_2_-atmosphere glovebox. To remove unreacted SnCl_2_ and any reaction byproducts, the V_2_O_5_ dispersion was centrifuged in the glovebox at 3000 rpm for 30 s, and the supernatant was poured off. Neat, dry methanol was added to the V_2_O_5_ nanoparticles and shaken up to redisperse. The process was repeated at least 3 times to observe minimal Cl 2p signal in XPS measurements. Mesoporous films were immersed in the same SnCl_2_ solution. We avoided hydrolysis and oxidation side products by performing the SnCl_2_ reduction chemistry in a N_2_-atmosphere glovebox with solvents that are dry and stored over 3 Å molecular sieves.

### Transmission Electron Microscopy

TEM samples were prepared by drop-casting a small amount of untreated and treated V_2_O_5_ samples onto carbon-coated Cu TEM grids. TEM imaging and diffraction data were collected on an FEI Tecnai F20 S/TEM operating at 200 kV. High-resolution scanning (S)TEM imaging and energy dispersive X-ray spectroscopy (EDX) were performed on a Thermo Fisher Scientific (TFS, formerly FEI) Spectra200 STEM operating at 200 kV with a convergence semiangle of 24.7 mrad and equipped with a TFS Super-X EDX detector.

### X-ray Photoelectron Spectroscopy

V_2_O_5_ samples that were untreated and treated with SnCl_2_ were drop-cast onto Au-coated glass substrates inside a N_2_-atmosphere glovebox. Samples were transferred air-free from the glovebox to the XPS system through a load-lock. XPS data were obtained on a PHI Versa Probe III instrument using Al Kα radiation (1486.7 eV). The XPS data were calibrated with Au and/or Cu metal, which was cleaned via Ar-ion sputtering. The raw atomic concentration has a 5% error due to surface inhomogeneities, surface roughness, literature sensitivity values for peak integration, and so on.

### Hard X-ray Photoelectron Spectroscopy (HAXPES)

HAXPES was performed at the National Institute of Standards and Technology beamline SST-2 of National Synchrotron Light Source II at Brookhaven National Laboratory. The elemental core levels and valence bands were measured by using a 2 keV photon energy, 500 eV pass energy, and a 0.05 eV step size with the analyzer axis oriented in parallel with the photoelectron polarization vector. The valence bands of the materials were also measured using a 5 keV incident photon energy, 200 eV pass energy, and a 0.05 eV step size. The photon energies were selected using a double Si (111) crystal monochromator, and the beam energy was aligned to the Fermi level of a gold foil prior to measurement. Commercial equipment, instruments, or materials are identified in this study to adequately specify the experimental procedure. Such identification is not intended to imply recommendation or endorsement by the National Institute of Standards and Technology, nor is it intended to imply that the materials or equipment identified are necessarily the best available for the purpose.

### X-ray Diffraction

XRD patterns were collected in air using a Bruker D8 Discover diffractometer with a GADDS four-circle detector (General Area Detector Diffraction System) and Cu Kα (1.5406 Å, 8.04 eV) radiation.

### Raman Spectroscopy

Raman spectroscopy was performed on a Renishaw inVia system (Gloucestershire, U.K.) by using a 532 nm laser and 100× magnification or 50× long working distance objective lenses. The scattering light from the sample was directed by a grating with 1800 lines mm^–1^.

### Electron Paramagnetic Spin Resonance (EPR) Spectroscopy

V_2_O_5_ samples that were untreated and treated with SnCl_2_ were prepared by adding V_2_O_5_ dispersions to a quartz (CFQ) 4 mm outer diameter tube. X-band (9.38 GHz) continuous-wave (CW) EPR measurements were performed on a Bruker ELEXSYS E500 spectrometer equipped with a super high-Q resonator, along with a cryogen-free temperature system (ColdEdge Technologies) and MercuryiTC temperature controller (Oxford Instruments) for low-temperature measurements. The Xepr software (Bruker) was used for data acquisition. Spectra were recorded at 60 K, using a modulation frequency of 100 kHz, a modulation amplitude of 10 G, and a microwave power of 0.1 mW.

### UV–Visible–Near-Infrared Absorbance Spectroscopy

UV–vis–NIR absorbance measurements were performed on a Cary 5000 spectrophotometer equipped with a diffuse reflectance accessory with a photomultiplier tube and PbS detectors (DRA-2500). The sample was mounted at the center of the DRA-2500 integrating sphere, using a variable angle center mount holder, so that the measurement compensated for specular and diffuse reflectance/scattering from the sample. Baselines for 0 and 100% transmission were collected prior to measurement of the samples.

### Dark Microwave Conductivity

Dark microwave conductivity measurements were performed in a custom-designed X-band copper microwave cavity, as described previously.^70^ To calculate the conductance of each sample for the microwave measurement, we used the commercially available COMSOL Multiphysics (v4.3) finite element package to solve Maxwell’s equations for the electromagnetic field distribution within the cavity, as described in detail elsewhere.^73^ Replicates of each measurement were performed to obtain statistically meaningful average values and standard deviations.

### V_2_O_5_ Device Fabrication

To fabricate the device, a commercially available glass substrate was used, followed by a cleaning process involving soap/DI water, acetone, and IPA. The α-V_2_O_5_ film was then deposited using the spin-coating method at 4000 rpm for 60 s, with a film thickness of approximately 2.6 μm (Figure S7). The glass/V_2_O_5_ film stack underwent annealing in an oven for 30 min at 500 °C under ambient conditions. Finally, a top metal contact (Au) was deposited using thermal deposition with a shadow mask, resulting in a thickness of 100 nm. The overall device configuration features six 0.1225 cm^2^ device pixels on each substrate.

### Electrical Transport Measurement

Electrical performance of our two-terminal devices was evaluated using a Keithley 4200 parameter analyzer by performing current–voltage (*I*–*V*) scans in ambient conditions at room temperature. The specified voltage bias (from the source electrode to the drain electrode) was applied to the device in the following sequence: −10 V → 0 V → 10 V. Four-terminal van der Pauw measurements were performed on samples prepared with dimensions of 1 cm × 1 cm. The measurements were performed using the M91 FastHall measurement controller in ambient conditions at room temperature, with a 1 T magnetic field applied and an excitation source current of 10 mA during the experiment.

### Theory

We employed quasiparticle self-consistent GW (QSGW) approximation,^67^ a many-body perturbative approach, which is a self-consistent form of Hedin’s GW approximation.^74^ Self-consistency eliminates the starting point dependence, and as a result, the discrepancies are much more systematic than conventional forms of GW.^75^ Further, the excitonic correlations are included within a self-consistent extension of QSGW, called QSGŴ,^76^ where the electron–hole excitonic effects are taken into account by solving the Bethe–Salpeter equation in the presence of electron–hole ladder corrections. The QSGŴ is also fully self-consistent; the electronic eigenfunctions are recomputed in the presence of these excitonic correlations and vice versa, until self-energy, Green’s functions, and optical polarizability, all of them achieve self-consistent convergence with desired accuracies. The importance of self-consistency in both QSGW and QSGŴ for different materials has been explored.^77^ The theory and its application to a large number of both weakly and strongly correlated insulators is given in ref (76). To simulate the different kinds of vacancies, we prepared supercells of sizes 42, 56, and 84 atoms. The electronic and optical properties of the 56- and 84-atom supercells are very similar, and all qualitative conclusions for the two are same. We present the results for the 84-atom supercell in the present study.

## Supporting Information Available

Core-level hard X-ray photoelectron spectroscopy (HAXPES) for Sn^4+^ 3d_3/2_ and 3d_5/2_ and Cl 2p peaks; valence band HAXPES measured at 5 eV; electron diffraction patterns for untreated α-V_2_O_5_ and α-V_2_O_5_ treated with 0.05 M SnCl_2_; Raman spectroscopy of V_2_O_5_ nanoparticles treated with different concentrations of SnCl_2_; XRD diffraction patterns of V_2_O_5_ nanoparticles treated with different concentrations of SnCl_2_ in acetone for 90 min; optical profilometry images of α-V_2_O_5_ films; stoichiometry from XPS derivation. (PDF)
