# Supplementary material for: Tuning Optical and Electrical Properties of Vanadium Oxide with Topochemical Reduction and Substitutional Tin
Source: Chem Mater. 2024 Oct 17;36(21):10483–95. doi: 10.1021/acs.chemmater.4c01557 (PMC11562072; doi:10.1021/acs.chemmater.4c01557)
Supplement: Supplementary file 1 — cm4c01557_si_001.pdf [file cm4c01557_si_001.pdf]

Supporting Information:

Tuning Optical and Electrical Properties of Vanadium Oxide with Topochemical Reduction and Substitutional Tin

Lance M. Wheeler,[1]* Thanh Luan Phan,[1] Michelle A. Smeaton,[1] Swagata Acharya,[1] Shruti Hariyani,[2] Marlena E. Alexander,[1] Miranda I. Gonzalez,[1] Elisa M. Miller,[1] David W. Mulder,[1] Sarbajit Banerjee,[2] Katherine L. Jungjohann,[1] Andrew J. Ferguson,[1]* Jeffrey L. Blackburn[1]*

[1]National Renewable Energy Laboratory, Golden, CO 80401, USA
[2]Department of Chemistry, Texas A&M University, College Station, TX 77843, USA

*Correspondence: Lance.Wheeler@nrel.gov, Jeffrey.Blackburn@nrel.gov, Andrew.Ferguson@nrel.gov

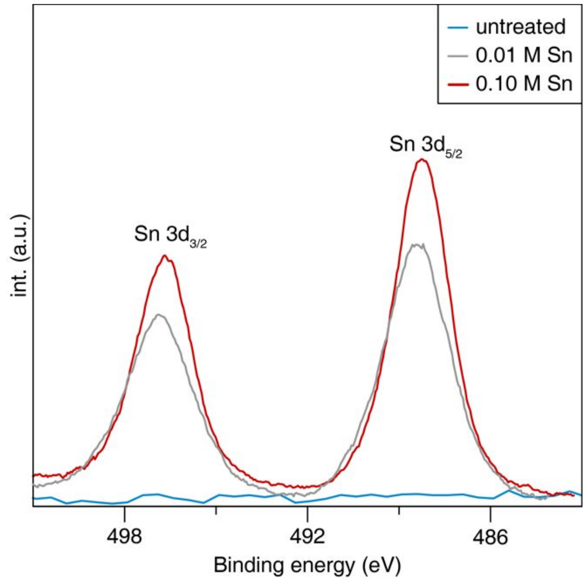

Figure S1: Core-level hard X-ray photoelectron spectroscopy (HAXPES) Sn^{4+} $3d_{3/2}$ and $3d_{5/2}$ peaks. The intensity of the Sn^{4+} $3d_{3/2}$ and $3d_{5/2}$ peaks also increase upon treatment of V_2O_5 with higher concentrations of $SnCl_2$, suggesting increased substitution of Sn^{4+}, as expected. The excitation energy is 2 keV.

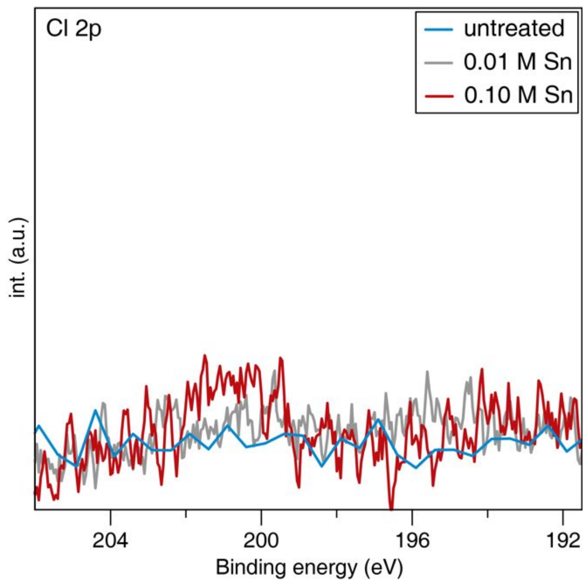

Figure S2: Core-level hard X-ray photoelectron spectroscopy (HAXPES) of the Cl 2p peak for untreated V_2O_5 and V_2O_5 treated with 0.01 M $SnCl_2$ and 0.10 M $SnCl_2$. The excitation energy is 2 keV.

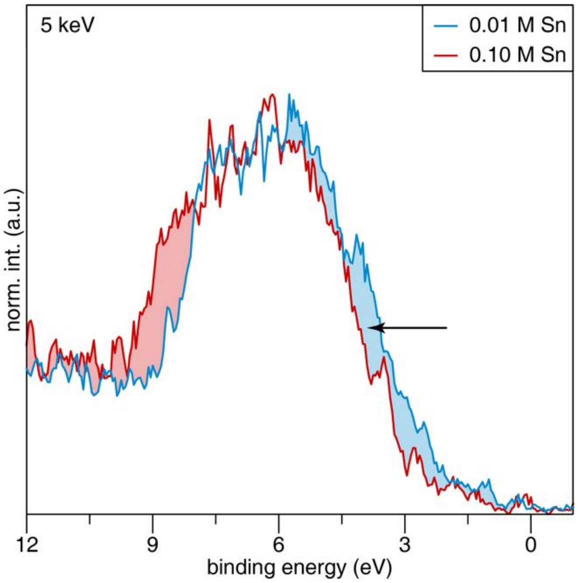

Figure S3: Valence band hard X-ray photoelectron spectroscopy (HAXPES) data measured at 5 keV.

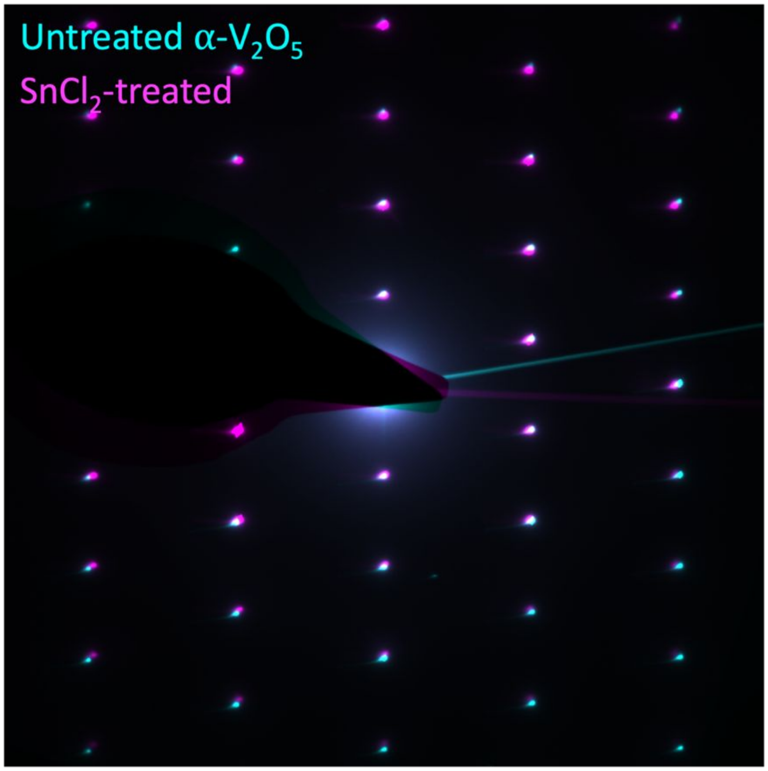

Figure S4: Electron diffraction patterns for untreated α-V_2O_5 and α- V_2O_5 treated with 0.05 M $SnCl_2$.

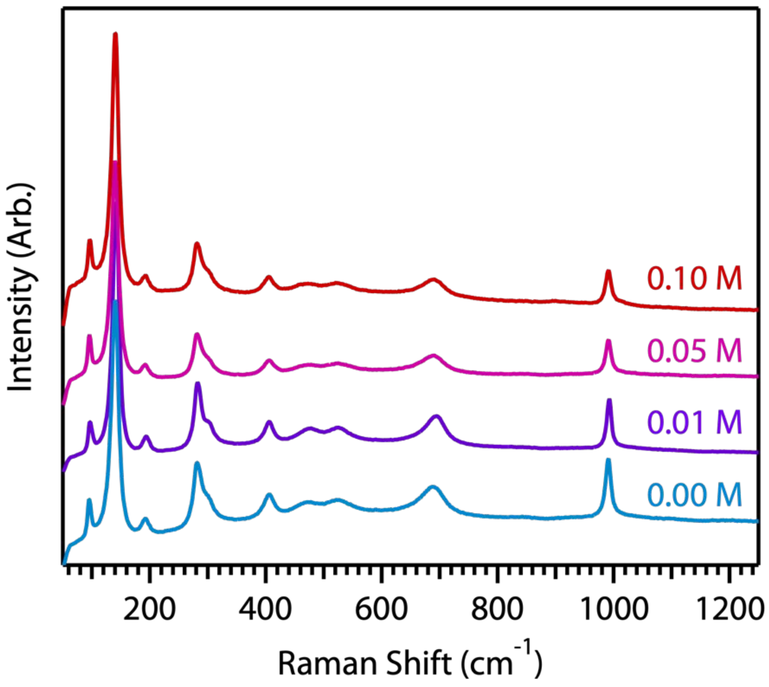

Figure S5: Raman spectroscopy of V_2O_5 nanoparticles treated with different concentrations of $SnCl_2$ in methanol for 90 minutes.

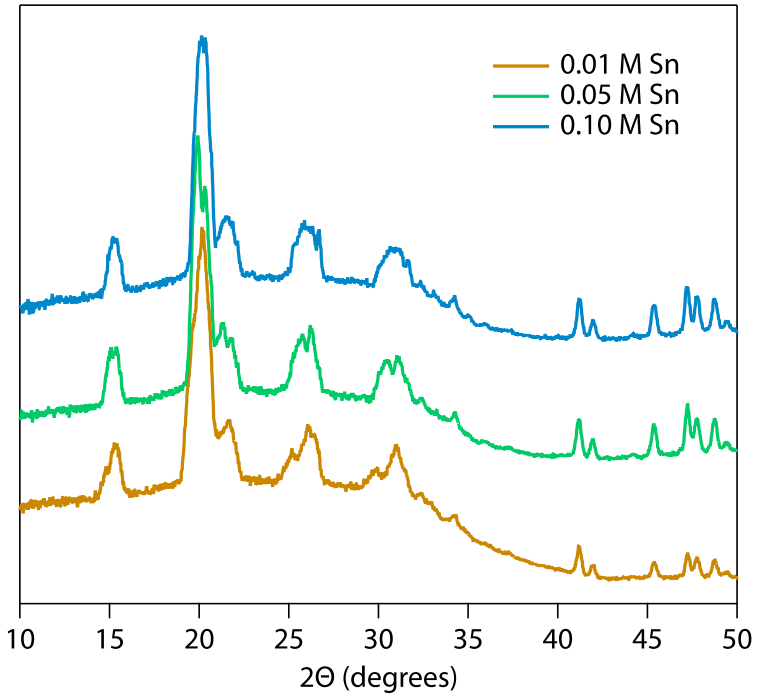

Figure S6: XRD diffraction patterns of V_2O_5 nanoparticles treated with different concentrations of $SnCl_2$ in acetone for 90 minutes.

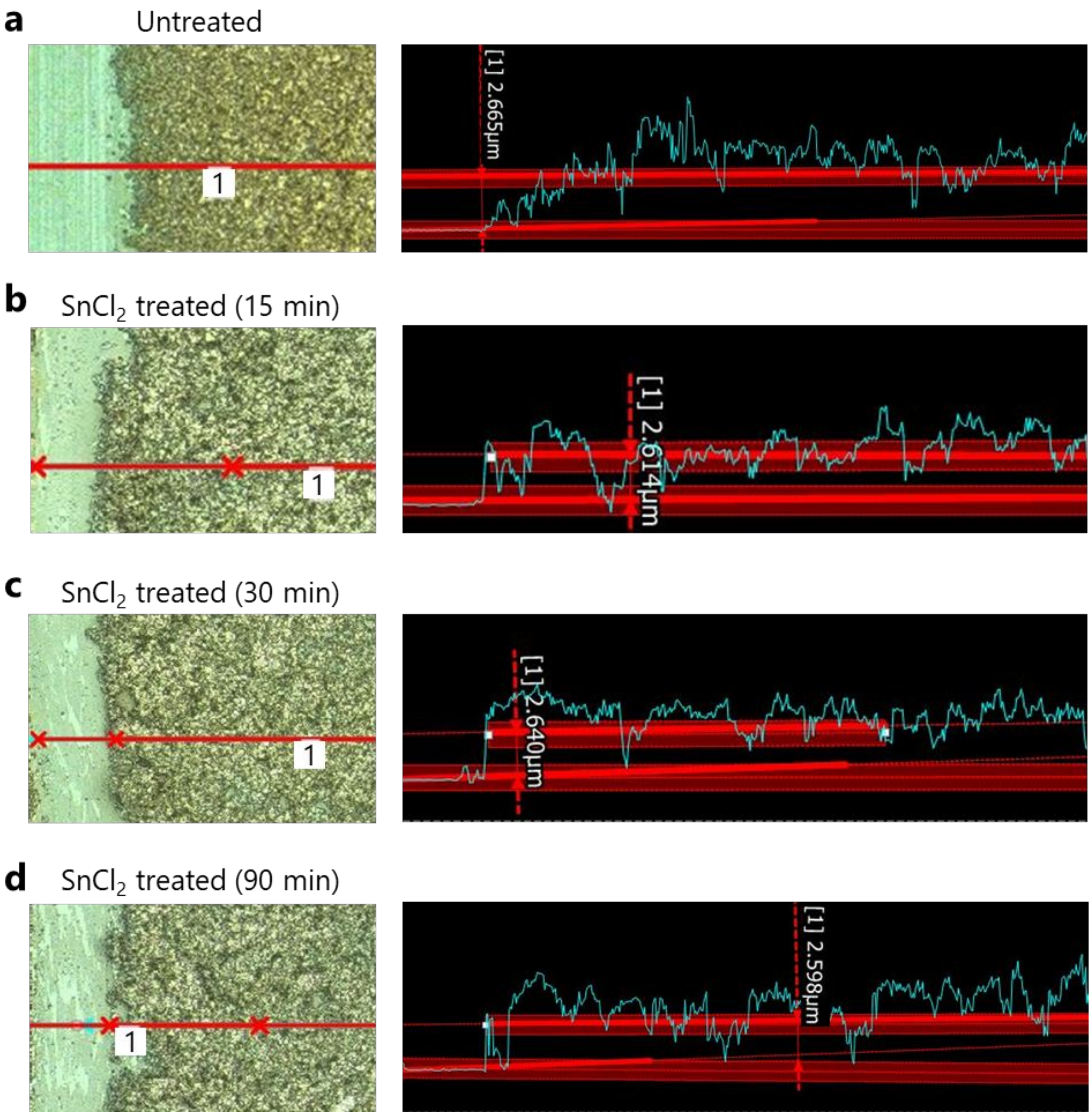

Figure S7: Optical profilometry images (left row) and height profiles (right row) of V_2O_5 samples. **a)** Untreated V_2O_5, and $SnCl_2$-treated V_2O_5 at treatment durations of **(b)** 15 minutes, **(c)** 30 minutes, and **(d)** 90 minutes. Measurements were conducted using a KEYENCE VHX 3D Profiler Measurement Module for VHX-7000. The thickness of the mesoporous film shows only a slight variation before and after $SnCl_2$ treatment, decreasing from 2.665 µm in the untreated sample to 2.614 µm after 15 minutes of treatment, 2.640 µm after 30 minutes, and 2.598 µm after 90 minutes.

Stoichiometry from XPS derivation:

Reaction assuming starting with stoichiometric V_2O_5 (for reference):

$$xSn^{II}Cl_2 + V_2^VO_5 \longrightarrow (x-2xy)Sn^{IV}OCl_2 + (2xy)V^{IV}OCl_2 + \left(Sn_y^{IV} + V_{1-y}^{IV}\right)_{2x}V_{2-2x}^VO_{5-x}$$

Stoichiometry for individual elements:

$$xSn^{II} \longrightarrow xSn^{IV} - 2xySn^{IV} + 2xySn^{IV} + 2xe^-$$

$$2V^V + 2xe^- \longrightarrow 2xyV^{IV} + 2xV^{IV} - 2xyV^{IV} + 2V^V - 2xV^V$$

$$5O \longrightarrow xO - 2xyO + 2xyO + 5O - xO$$

$$2xCl \longrightarrow 2xCl - 4xyCl + 4xyCl$$

Reaction assuming starting with non-stoichiometric V_2O_5:

$$xSn^{II}Cl_2 + V_{2a}^{IV}V_{2-2a}^VO_{5-a} \longrightarrow (x-2xy)Sn^{IV}OCl_2 + (2xy)V^{IV}OCl_2 + \left(Sn_y^{IV} + V_{1-y}^{IV}\right)_{2x}V_{2a}^{IV}V_{2-2a-2x}^VO_{5-a-x}$$

Stoichiometry for individual elements:

$$xSn^{II} \longrightarrow xSn^{IV} - 2xySn^{IV} + 2xySn^{IV} + 2xe^-$$

$$2V^V - 2aV^V + 2aV^{IV} + 2xe^- \longrightarrow 2xyV^{IV} + 2xV^{IV} - 2xyV^{IV} + 2aV^{IV} + 2V^V - 2aV^V - 2xV^V$$

$$5O - aO \longrightarrow xO - 2xyO + 2xyO + 5O - aO - xO$$

$$2xCl \longrightarrow 2xCl - 4xyCl + 4xyCl$$

Ratio of V^{IV} to V^V (from XPS of starting material):

$$\frac{V^{IV}}{V^V} = \frac{2a}{2-2a}$$

$$2a = (2-2a)\frac{V^{IV}}{V^V} = 2\frac{V^{IV}}{V^V} - 2a\frac{V^{IV}}{V^V}$$

$$a + a\frac{V^{IV}}{V^V} = \frac{V^{IV}}{V^V}$$

$$a\left(1 + \frac{V^{IV}}{V^V}\right) = \frac{V^{IV}}{V^V}$$

$$a = \frac{\frac{V^{IV}}{V^V}}{\left(1 + \frac{V^{IV}}{V^V}\right)}$$

Ratio of Sn^{IV} to total M (from XPS of doped material) – assumes complete removal of oxychlorides:

$$\frac{Sn^{IV}}{M} = \frac{2xy}{2xy + 2x - 2xy + 2a + 2 - 2a - 2x} = \frac{2xy}{2} = xy$$

Ratio of Sn^{IV} to V^{IV} (from XPS of doped material) – assumes complete removal of oxychlorides:

$$\frac{Sn^{IV}}{V^{IV}} = \frac{2xy}{2x - 2xy + 2a}$$

$$2xy = (2x - 2xy + 2a)\frac{Sn^{IV}}{V^{IV}} = 2x\frac{Sn^{IV}}{V^{IV}} - 2xy\frac{Sn^{IV}}{V^{IV}} + 2a\frac{Sn^{IV}}{V^{IV}}$$

$$xy = x\frac{Sn^{IV}}{V^{IV}} - xy\frac{Sn^{IV}}{V^{IV}} + a\frac{Sn^{IV}}{V^{IV}}$$

$$x\frac{Sn^{IV}}{V^{IV}} = xy + xy\frac{Sn^{IV}}{V^{IV}} - a\frac{Sn^{IV}}{V^{IV}}$$

$$x = \frac{xy + xy\frac{Sn^{IV}}{V^{IV}} - a\frac{Sn^{IV}}{V^{IV}}}{\frac{Sn^{IV}}{V^{IV}}}$$

$$x = \frac{\frac{Sn^{IV}}{M} + \frac{Sn^{IV}}{M}\frac{Sn^{IV}}{V^{IV}} - a\frac{Sn^{IV}}{V^{IV}}}{\frac{Sn^{IV}}{V^{IV}}}$$
